# Relational Autonomy, the Right to Reject Treatment, and Advance Directives in Japan

**DOI:** 10.1007/s41649-021-00191-1

**Published:** 2021-10-09

**Authors:** Anri Asagumo

**Affiliations:** Department for Continuing Education, University of Oxford, Oxford, UK

**Keywords:** Relational autonomy, The right to reject treatment, End-of-life decision-making, Advance directives

## Abstract

Although the patient’s right to decide what they want for themselves, which is encompassed in the notion of ‘patient-centred medicine’ and ‘informed consent’, is widely recognised and emphasised in Japan, there remain grave problems when it comes to respecting the wishes of the no-longer-competent when death is imminent. In general, it is believed that the concepts above do not include the right to refuse treatment when treatment withdrawal inevitably results in death, even when the patient previously expressed the wish to exercise this right when competent. In this paper, I first explain the current social and legal situation in Japan, where the lack of legal clarity regarding the right to reject treatment tends to result in doctors adopting the interpretation of patients’ words that is least conducive to treatment withdrawal. I then argue that the right to refuse treatment should be taken seriously, even when the patient is no longer competent, or the treatment refusal will result in death. I suggest that the concept of relational autonomy might have some practical and valuable implications in a country where individual autonomy is considered incompatible with societal values. Finally, I answer possible objections to relational autonomy and address the widespread societal concern about sliding down the slippery slope from allowing the right to refuse treatment to the obligation to die.

## Patients’ Neglected Right to Reject Treatment

While the right to refuse treatment is now widely recognised in western countries, it is still controversial whether it is legally and ethically permissible to withdraw life-sustaining treatment in Japan at the patient’s request. Although there have not been any cases in which doctors have been charged solely for withholding or withdrawing artificial ventilation, doctors are reluctant to do so for fear of prosecution (Higuchi 2018). In Japan, the right to reject treatment is normally understood to exclude cases where treatment withdrawal will inevitably result in death, even if withdrawal in such a case would comply with an advance directive. The reluctance to countenance treatment withdrawal derives from confusion and dispute regarding the widely acknowledged concept of ‘*songenshi*’, as well as the lack of clear legal statements that protect doctors from the charge of murder.

*Songenshi* is a Japanese word that can be approximately translated as ‘death with dignity’ or ‘natural death’. It is typically contrasted with attempts to extend life at any cost. It is generally taken to mean death without life-prolonging treatment or being attached to machines, although this is not clearly stated anywhere (Kai 2012). The Japan Society for Dying with Dignity, which used to have euthanasia in its name, started to use the term ‘*songenshi*’ in 1981 to coincide with the World Medical Association’s Declaration of Lisbon on the rights of the patient, which adopts the term ‘death with dignity’. *Songenshi* is normally invoked either in relation to passive euthanasia or exercising the right to reject treatment. However, in Japan, it is still common to see doctors disrespecting patients’ wishes regarding *songenshi*, for example the wish to have artificial ventilation withdrawn, for fear of being prosecuted for murder (Higuchi 2015). This is partly because, in 2008, two doctors were almost indicted for murder after removing respirators from seven patients with terminal cancer on request of the patients’ families. Although they were acquitted of the charges, doctors remain wary of the legal risks.

Although a clear legal statement is yet to be made, there are several guidelines on end-of-life decision-making, including on the withholding/withdrawal of certain kinds of treatments (Ministry of Health, Labour and Welfare 2018b; Japanese Society of Intensive Care Medicine et al. 2014). The common theme in these guidelines is that they treat the *process* of decision-making as more important than reaching a decision or the nature of the decision. This may reflect the negative attitudes held by citizens and medical professionals on the documentation of decisions concerning death.

The attitude of doctors erring on the side of caution and their aversion to documentation seem to manifest a failure to respect individual autonomy. Ethicists who believe in the supreme value of individual autonomy might say that failure to comply with individual wishes is an indication of doctors acting immorally. However, I would argue that the Japanese approach to autonomy suggests that this does not necessarily follow. On the contrary, encouraging individual autonomy in Japan without a relational perspective could result in more harm than good, as I argue in the ‘Possible Objections’ section. In the ‘The Right to Reject Treatment Should Be Taken Seriously’ section, I argue that a better response than emphasising individual autonomy would be to advocate relational autonomy in balancing benefits and harms regarding the right to reject treatment. In the ‘The Concept of Relational Autonomy’ section, I introduce the concept of autonomy from a relational perspective and illustrate how it helps to respect patients’ wishes in Japan. In the ‘Possible Objections’ section, I respond to the possible objections.

## The Right to Reject Treatment should be Taken Seriously

In most cases, medical interventions are administered with the aim of enhancing our well-being. Individual autonomy is important when making decisions in the medical context in part because it helps us decide what matters the most to our well-being. Often, health is not the priority that surpasses everything else. Instead, we sometimes trade off health against other goods. Some engage in potentially life-risking recreational pursuits, for example, while others take substances harmful to their health for pleasure. This suggests that we value health only because it can serve us to pursue other goods in life.

In the medical context, the principle of beneficence is often understood to be grounded by a narrow objectivist understanding of the principle of beneficence that prioritises health over other goals. This is in contrast with a broader conception of beneficence, with which the principle takes into account ‘*any* prudential benefits, without assigning any particular weight to a specific category of those benefits.’ (Pugh 2020, 253).

To illustrate, imagine the following case, which resembles one that I experienced while working as a junior doctor, but with details altered to preserve anonymity. Mr Yamada, an 83-year-old man with moderate dementia, was taken to hospital by ambulance with a condition that required intubation and intensive care treatment. A few days before the event, he had visited the hospital with his family for another reason, but the medical record indicated that he stubbornly declined any sort of intervention. Besides that, he had a note, written before he developed dementia, expressing his general distrust in hospitals and doctors and his desire to avoid life-prolonging treatment. His family testified that his thoughts had not changed over the intervening years. However, he eventually underwent all the standard treatments for patients in his condition. His wife, who called an ambulance in panic, regretted her decision, and asked the doctors to stop all interventions, acknowledging that it meant Mr Yamada’s death.

The ethics committee denied the request by appealing to a narrow objectivist understanding of the principle of beneficence, with reference to the ambiguous descriptions in law. In addition, they argued that the wishes expressed in writing where invalid on the grounds that they were written more than ten years before and did not identify specific medical conditions, and that his most recent expression of wishes was the nod he gave to his family just before losing consciousness when they frantically convinced him to accept treatments.^1^

As can be seen in the case of Mr Yamada, it seems that at least three factors explain reluctance to respect the wishes of patients in withdrawing or withholding treatment: uncertainty about the patient’s autonomy with respect to his refusal, the aforementioned narrow interpretation of what the principle of beneficence demands in the context, and the fear of legal prosecution that I mentioned earlier. Given the invalidity of the advance directives, individual autonomy is not deemed applicable in cases like Mr Yamada’s, and the narrow interpretation of beneficence is adopted, which regards death as not being in a person’s interests (Pugh 2020).

However, it should be noted that even when there is no need to comprehend highly complex information, we can make decisions with grave consequences (Pugh 2020). For example, refusing a blood transfusion despite massive bleeding could lead to death, and it is not difficult to understand the worst-case scenario arising from the decision. What Mr Yamada held was this type of belief. If an individual decides to weigh up something over health in their balancing of goods, I believe that beneficence requires us to respect this.

When the will of the patient has been clearly stated in the past or the patient insists on non-treatment using their language, and their significant others can confirm it, the right to reject treatment should be taken much more seriously than it currently is. Moreover, it should not be required that the patient fully understand all the possible diseases they could contract and possible outcomes of treatments. Even the western idea of informed consent requires only a substantial understanding of information that is material to their decision. Very specific information about the precise diagnosis and possible outcomes of treatments for which they must make an advance directive would probably not qualify as material to the decision to reject certain sorts of treatment. Therefore, there should be no problem acting in accordance with patients’ wishes even if it results in death if the patient was able to broadly sketch the circumstances and reasons for which they do not wish to receive treatment.

### The Necessity of Legislation and the Normative Theory Underpinning the Guidelines

There remains a wary opposition among doctors and the general public in Japan to respecting the right to reject life-saving treatment. Some statistics suggest that there is reluctance among not only the general public but also doctors to facilitate formal advance care planning (Ministry of Health, Labour and Welfare 2018a). However, the hesitance of the majority to leave or facilitate advance directives does not justify the lack of a legal framework, and stagnation on the issue gives rise to the possible necessity of interpreting the concept of autonomy differently to accommodate dilemmas in clinical settings in Japan.

In a situation like Mr Yamada’s, people trust doctors and their significant others to carry out their wishes. However, this trust is often misplaced in the current system. The reluctance of doctors and patients to leave a written advance directive may reflect people’s concerns about a possible change in the situation that would affect their way of thinking, something unexpected happening in the future, or the process becoming a banal, tick box exercise (Gómez-Vírseda et al. 2019). Whatever reason they may have, the current landscape, where doctors are afraid of legal charges and, as a result, cannot withhold or discontinue treatment (Kondo and Mikami 2018), needs to be changed to ensure that people’s wishes are respected.

It is also possible that doctors feel guilty about not initiating or withdrawing treatment; at least a decade ago, a commonly held view among senior doctors was that not providing a gastric tube is akin to starving patients to death, for example (Aita 2012). Relational autonomy could alleviate this kind of psychological conflict, as I describe later.

As such, the lack of legal clarity regarding the right to reject, withdraw, or withhold treatment tends to result in doctors adopting the interpretation of patients’ words that are least conducive to treatment withdrawal. This results in their failure to fulfil what the patient would have wanted. Therefore, doctors need better legal protection where they adhere to advance directives. In addition to legal reforms designed to respect advance directives better, I suggest that a more fundamental rethinking of autonomy with greater attention given to the concept of relationality might help to facilitate better understanding and realisation of advance directives in Japan.

## The Concept of Relational Autonomy

Relational autonomy denotes various perspectives that understand autonomy from a relational standpoint. It attempts to accommodate the complexities we face in clinical practice, which cannot be well captured through the individualistic, atomistic approaches to autonomy (Mackenzie and Stoljar 2000). According to a 2019 systematic review (Gómez-Vírseda et al. 2019), relational autonomy is ‘a ‘reaction against’ an individualistic interpretation of autonomy’, so the concept does not have a single definition or philosophical framework it relies on, but the critical element of it is a dialogue. Thus, even though there is no consensus on a normative concept yet, the accounts of relational autonomy emphasise the importance of dialogue to embrace diversity in our pluralistic world.

Based on this systematic review, Gómez-Vírseda et al. (2020, 3-5) identify four shortcomings of the traditional understanding of autonomy from which a key understanding of relational autonomy can be developed: *autonomy entails more than merely possessing cognitive capacity; autonomy is not exercised by patients existing in a social and cultural void; autonomy is not a binary ‘all-or-nothing’ condition; autonomy is not exercised in terms of isolated discrete discussions*. In this paper, I use the following definition of relational autonomy proposed by Gómez-Vírseda et al. (2020, 6-12): Autonomy is a multidimensional capacity which consists of emotions and bodily mediated experiences besides rationality; autonomy is exercised in a sociocultural context that shapes us, and the relationships between patients, family, and personal relationships, and healthcare professionals are able to enhance or undermine autonomy; for these reasons, autonomy manifests itself in a scale, and we can be more or less autonomous rather than be or not be autonomous; therefore, autonomy is a temporal perspective evolving and unfolding over time through interactions with others. I argue that it would be beneficial to introduce this analysis of autonomy into clinical practice in Japan. I defend my view in relation to the second, third, and fourth points of the shortcomings of individual autonomy suggested by Gómez-Vírseda et al.

### Acknowledgement of the Values that are Material to Decision-making

Relational autonomy helps us better understand what patients value in their lives by acknowledging that their relationships matter to them. Most of us would not assert that the best decision for ourselves is necessarily that which benefits ourselves most. Most of us live in an intricate web of relationships, and it is not uncommon for us to choose an option that benefits someone close to us, even if we incur losses as a result. As Ho (2008, 131) notes, ‘perhaps more important to them is the preservation of overall sense of identity, agency, and selfhood through connections with others’ than the exercise of autonomous deliberation. Examining patients’ wishes in light of individual autonomy, which solely relies on the individual patient’s interests, discounts the kind of intricacy from which we form our desires. It is not strange at all when someone thinks that she wants a certain option *as long as* it does not significantly reduce the well-being of her family. Indeed, empirical research suggests that for many Japanese patients, ‘to make a decision on medical treatment is to reach an agreement between the patient, the physician, and the patient’s family’ and the independence of the opinion from their family does not normally matter, for they do not usually think that the interests of themselves and their families conflict (Sekimoto et al. 2004, 6).

I mentioned earlier that Mr Yamada’s rejection of medical intervention came from his distrust of hospitals and doctors, but he also had some notes on his wishes not to be a ‘burden’ to his wife by being bed-bound and entirely relying on her. Mr Yamada was firmly convinced that it was better for him to die than be bed-bound, but it is difficult for many people to assert something about their uncertain future. The wish to not be a burden to their family is very common among elderly people when we ask about their future plans (Broom and Kirby 2013; Cahill et al. 2009; Sandsdalen et al. 2015). An emphasis on relational autonomy can reassure such patients as they know that the final decisions on their treatment would be made based on the overall welfare of them and their family in line with their wishes.

### Scaler Notion Suits Better for End-of-Life Decision-making

Relational autonomy acknowledges that autonomy fluctuates ‘across the time and sphere of our lives’ due to our social embeddedness (Nedelsky 2012). This understanding of autonomy is practical in end-of-life decision-making because physical and emotional distress affects decision-making capacity (Gómez-Vírseda et al. 2020). Decision-making is not always straightforward, and one reason is that the process is not always rational (Lolich and Lynch 2017; Walter and Ross 2014). When decision-making capacity is compromised to some degree, assistance from their loved ones is helpful to evaluate whether the decisions patients make align with what they genuinely value (Tonelli and Misak 2010). This is particularly the case with people with dementia (Głos 2016).

Relational autonomy is compatible with the decision-making process being ‘fundamentally dynamic, continually constructed and reconstructed in dialogic processes with other people’ (Walter and Ross 2014, S19). Dialogue between patients, family, their loved ones, and healthcare professionals shapes patients’ preferences and values over time. This understanding does not force people to reach a conclusion in haste and allows changes in their decisions whenever possible if they later reach a preferable, reconsidered alternative in light of a patient’s and their loved ones’ wishes (Gómez-Vírseda et al. 2020).

Even though rationality and memory are impaired in patients with dementia, they display their preferences and crude rationality hidden in and tangled with emotions. Close relationships with family and friends can help them express themselves and maintain their sense of self by constituting part of their identity, shaped and restored through relationships (Głos 2016). For this reason, in my opinion, discussions between patients, family, and medical professionals facilitate relational autonomy, and doctors can take wishes of patients with dementia as authoritative as long as these are discernible from their previous interactions and lifestyles, believing families as witnesses. Mr Yamada developed dementia, which slowly but steadily affected his mind and body, but his closeness to his wife and son had been the same over the decades. As long as there is no significant change in the relationship between them, I would trust the testimony from the family even if patients can no longer express themselves.

The turning point of Mr Yamada’s case was that his wife called an ambulance in panic even though she knew that Mr Yamada would not want any life-extending interventions. She then decided in haste that she wanted him to be intubated when asked by an emergency doctor in a stressful environment with little time left for her to decide. What changed her mind later was her husband’s subsequent physical status, from being very sick to being intubated (‘sicker’ in her understanding). Only then did she realise that it was not what either she or her husband wanted. Often, a severely demented patient’s most available surrogate decision-maker may also be mildly or moderately demented.^2^ It is hard, even for people without cognitive impairments, to develop their family’s treatment plans. It is harder to obtain a meaningful, fully informed decision from people with cognitive impairments in a short window of time. Naturally, they change their mind when confronted with ones’ conditions and the explanations on the situation by medical staff in a way they can understand. The explanation usually takes time and requires a good understanding of patients’ and their family’s personalities.

For these reasons, the default understanding of autonomy should be from the relational perspective so that patients and their loved ones can take time to understand the situation and make a deliberate decision in their own way, on end-of-life issues. Relational autonomy can allow room for the feelings which make them dither when rationality cannot play a role. The visual aid of Mr Yamada’s ‘sicker’ condition and the input from medical professionals in an understandable way was necessary for Mr Yamada’s wife to assimilate Mr Yamada’s view with her temporal wish which was driven by fluctuating emotions under a stressful environment.

### Cultural Affinity

Giving more weight to discussions within the contexts of patients’ important relationships requires understanding the nuances and connotations of the language patients have developed throughout their life. For example, when one asks, ‘What is the meaning of life?’ to an elderly patient in England, one may answer their sense of mission or what they weigh and cherish in their life. However, to elicit a similar answer from a Japanese patient, we do not use expressions like ‘the meaning of life’ or ‘the purpose of life’; these terms are not used in daily Japanese. Instead, one might ask, ‘What were the memorable episodes in your life?’ Thus, personal values cannot be bundled into specific categories based on literal interpretation but can only be grasped through someone close to the patient for decades (Becker 2019).

Another cultural factor to consider is the concept of ‘heart to heart communication’ (*ishin denshin*), which indicates a tacit understanding of others without words (Cheung 2018). ‘*Sasshi’* is a similar concept which means a type of communication based on the use and interpretation of non-verbal signs to convey or infer the intentions or wants (Nishida 1979). Vocal reticence in Japan is a form of social discretion and is taken as a sign of socially valued qualities such as modesty, politeness, and empathy (Lebra 2007). The sensitivity to non-verbal cues and ensuing responsiveness have allowed people within the culture to communicate in ‘attentive’ silence (Cheung 2018). With this cultural background, some people prefer extreme euphemism hoping that their non-verbal signs are well received, while others may lack the vocabulary to adequately or accurately describe what they want or feel. Nakazato et al. (2018) found that this inclination not to verbalise feelings tends to be preserved at the end of life, and their preference for ‘heart to heart communication’ may increase. However, the difficulty lies in the fact that cues and signs may be understood differently depending on the relationships between them and the aptitude of the receiver (Nishida 1979).

Whatever the reasons behind the difficulty of verbalising feelings are, understanding true intentions requires a deep understanding of and sensitivity to their characters and their everyday use of language. Traditional accounts of individual autonomy overlook such nuances, especially in cultures where verbal self-assertion is discouraged or a Western sense of individual autonomy is counterintuitive. Thus, relational autonomy allows more substantive input from the family rather than just looking at facts. Therefore, attempting to understand patients holistically through dialogue, physicians may feel less uncomfortable withholding or withdrawing life-sustaining treatment.

## Possible Objections

### Could the Right to Reject Treatment Turn into the Obligation to Reject Treatment?

Opponents to the relational approaches to autonomy cite the danger of coercion. Tamura (2006) points out that modesty and mindfulness of the feelings of others are considered to be more important than the idea of self-esteem or assertiveness in Japan, which could put pressure on patients internally and externally to prioritise the opinions of family members. Similarly, self-sacrifice is a key moral virtue in Japanese culture. Although everyday selfless acts to achieve social harmony are mostly harmless, there is a risk of patients choosing death to bring about a harmonious whole (Young 2002). Particularly worrisome may be the general public’s opinion, which could have a grave influence on individuals (Hongo 2014). Kodama (2019) suggests the right to reject treatment seems to be a slippery slope akin to eugenics which aims to solve the problems arising from rapid ageing and ballooning medical expenses.

For the concerns regarding coercion, I have two responses based on empirical and rational grounds, which further emphasise the importance of relational perspectives. Empirically, there is no evidence that an elderly population, considered socially disadvantaged, would suffer significantly due to the implementation of the right to reject treatment (Battin et al. 2007). Rationally, the solution to this objection would not be an endorsement of the myth of individual autonomy. Even in the context of individual autonomy, patients are as prone to external influences, including their doctors’, as they might be in relational autonomy. In a real-life setting, it is impossible to separate one’s preference from the environment one is in. Refusal to recognise the right to reject treatment for the reasons of embeddedness in a culture where modesty, mindfulness to others, and self-sacrifice are promoted could be viewed as belittling and patronising the values people have lived with throughout their lives.

To illustrate, consider choosing death to avoid ‘becoming a burden’. In a society where ‘not becoming a burden’ is a significant moral norm, it does not seem that incongruous to have the desire to die so as not to be a burden to their loved ones. If people want to choose death over prolonged unconsciousness or decreased well-being that affects the relationship with their family and loved ones permanently, it needs to be assessed carefully but should not be shrugged off and people should not be forced to undergo treatment that they wish to opt out of.

It is important to enact safeguards to protect the vulnerable from coercion. We may provide protection from an oppressive or abusive relationship by holding discussions with patients in private about their values, relationship with their family, reasoning process, and concerns about their situation (Ho 2008). When a clear sign of abuse or disregard is not evident in the private conversation, it would be better to follow the patients’ expressed wishes as a default stance (Ho 2006). The relational approach to autonomy I described above should help the medical staff be certain that the decision is in line with a patient’s true intention.

Moreover, the bedside is not the best place to combat social norms and institutions, even if those norms appear problematic (Ho 2008). Precisely because of the underlying culture, we need more acceptance of the idea of withholding or withdrawing treatments through relational approaches rather than avoiding them altogether for the danger of coercion.

### When the Wishes of Patients and Family Conflict, Which to Prioritise?

A patient’s opinion does not always coincide with the family’s opinion. Although prioritising patients’ wishes is the norm, the individualistic conception is at odds with the relational account of autonomy’s emphasis on the family unit as a whole and the harms that failing to respect treatment refusals causes to the unit in contrast to the individual. When there is a conflict between patients’ and family members’ wishes, I believe it is sometimes morally permissible or even desirable to endorse a family’s view.

As someone gets closer to their end of life, family and close people’s involvement is absolutely necessary if they want to fulfil certain kinds of wishes, such as their desire to be nursed at home and die at home. An example is ‘*rourou kaigo*’, care between elderly. *Rourou kaigo* is a burgeoning social problem in Japan (Hurst 2017). Among the shared-care households, about 60% of households reported that both carer and care-recipient were over 65 years old in 2020, and the percentage of households in which both parties were over 75 years old was around 33% (Ministry of Health Labour and Welfare 2019). It can happen when there is a monetary problem, limited space in a nursing home, or when some of the family members internalise a social stigma that putting their family into a nursing home is not an honourable thing to do, making them feel guilty about doing so (Hayashi 2011; Taylor et al. 2004). Under *rourou kaigo*, caregivers also tend to be in the phase of requiring care (Hurst 2017). Since nursing can be physically and mentally taxing, sometimes *rourou kaigo* leads to ‘*kaigo jigoku*’ (care-giving hell), subsequently neglect, abuse, or even homicide (Hayashi 2011).

There is a power relationship between physicians and lay patients as clinicians have specialised knowledge on diseases and treatments (Shih et al. 2018). When doctors endorse individual autonomy in the Japanese setting by giving paramount value to patients’ wishes, families may feel that they should suppress their feelings out of self-sacrifice to support the patients. As a result, during a patient’s last days, the family and patient may find their loving memories and close relationship replaced by emotional distress and, needless to say, the danger of *kaigo jigoku*.

One way to avoid this tragedy is to introduce relational autonomy, which encourages balancing the well-being and the needs of a patient, their family members and other loved ones, and the family as a whole (Walter and Ross 2014). When respecting patients’ wishes require their loved ones to make such great sacrifices that they are unable to have their own life, it should be acceptable to prioritise families’ opinions.

## General Implications and Lessons

The concept of relational autonomy can help us holistically understand patients’ wishes by paying attention to the relationships and environments that influence their decisions. It could also let family members’ opinions supersede patients’ wishes when the necessary condition is met. Although the right to reject treatment tends to be neglected in incapacitated patients in Japan, relational autonomy can provide good reasons for doctors to take the will of patients seriously, as does concern for patient well-being (i.e. beneficence). A change in the understanding of autonomy in medicine could pave the way for fulfilling patients’ wishes in Japan.

## Data Availability

Not applicable.

## Footnotes

1 Usually, consent is taken from the patient’s family when the patient does not have the capacity to consent, but there is disagreement among experts regarding the validity of conferring the right to decide on a patient’s family, and it is legally in a grey zone in medicine (Japan Federation of Bar Associations 2011, 5–6).

2 ‘*Ninnin kaigo*’ (care between people with dementia) often coincides with ‘*rourou kaigo*’* (care between elderly)*, which I describe later in the paper (Kumagai and Ishii-Kuntz 2016). Patients with dementia do not always have a fully competent surrogate decision-maker. In such a case, even when their partner or children also have some cognitive impairments, Japanese physicians tend to obtain consent from them (Masaki, Ishimoto, and Asai 2014). The misuse of and persistence in obtaining consent in Japan is outside the scope of this paper.

## References

[CR1] Aita Kaoruko (2012). Indications and values: PEG tube-feeding questions us how to achieve the best possible care for each patient from the viewpoint of clinical ethics. Journal of Japan Geriatrics Society.

[CR2] Battin Margaret P, van der Heide Agnes, Ganzini Linda, van der Wal Gerrit, Onwuteaka-Philipsen Bregje D (2007). Legal physician-assisted dying in Oregon and the Netherlands: Evidence concerning the impact on patients in “vulnerable” groups. Journal of Medical Ethics.

[CR3] Cahill Eileen, Lewis Lisa M, Barg Frances K, Bogner Hillary R (2009). You don’t want to burden them: Older adults’ views on family involvement in care. Journal of Family Nursing.

[CR4] Becker Carl (2019). Kango Ni Ikaseru Nihonjin No Shiseikan - Inishie Kara No Keisho [in Japanese]. Journal of Japan Association for Buddhist Nursing and Vihara Studies.

[CR5] Broom Alex, Kirby Emma (2013). The end of life and the family: Hospice patients’ views on dying as relational. Sociology of Health & Illness.

[CR6] Cheung, King-Kok. 2018. *Articulate Silences: Hisaye Yamamoto, Maxine Hong Kingston, and Joy Kogewa.* Ithaca, NY: Cornell University Press.

[CR7] Głos Aleksandra Małgorzata  (2016). Solidarity in healthcare - the challenge of dementia. Diametros.

[CR8] Gómez-Vírseda Carlos, De Maeseneer Yves, Gastmans Chris (2019). Relational autonomy: what does it mean and how is it used in end-of-life care? A systematic review of argument-based ethics literature. BMC Medical Ethics.

[CR9] Gómez-Vírseda Carlos, De Maeseneer Yves, Gastmans Chris (2020). Relational autonomy in end-of-life care ethics: A contextualized approach to real-life complexities. BMC Medical Ethics.

[CR10] Hayashi, Mayumi. 2011. The care of older people in Japan: myths and realities of family ‘care’. *History & Policy*, 3 June 2011. https://www.historyandpolicy.org/policy-papers/papers/the-care-of-older-people-in-japan-myths-and-realities-of-family-care. Accessed 13 September 2019.

[CR11] Higuchi Norio (2015). Legal issues on medical interventions in terminally ill patients. Medicine and Society.

[CR12] Higuchi, Norio. 2018. Withholding or withdrawing life-sustaining treatment [Japanese: 生命維持治療の差し控え、中止 ]. *Japan Medical Association Journal*, 31 August 2018. https://www.med.or.jp/doctor/rinri/i_rinri/c02.html. Accessed 25 October 2019.

[CR13] Ho Anita (2006). Family and informed consent in multicultural setting. American Journal of Bioethics.

[CR14] Ho Anita (2008). Relational autonomy or undue pressure? Family’s role in medical decision-making. Scandinavian Journal of Caring Sciences.

[CR15] Hongo, Jun. 2014. Euthanasia, the dilemma of choice. *Japan Times*, 15 February 2014. https://www.japantimes.co.jp/life/2014/02/15/general/euthanasia-the-dilemma-of-choice/. Accessed 5 September 2021.

[CR16] Hurst, Daniel. 2017. More than half of Japanese carers are pensioners. *The Times*, 28 June 2017. https://www.thetimes.co.uk/article/half-of-japanese-carers-are-pensioners-themselves-n2v3glbkz. Accessed 1 September 2021.

[CR17] Japan Federation of Bar Associations. 2011. Law Outline on Medical Consent Agency for Persons without Medical Consent Ability [Japanese: 医療同意能力がない者の医療同意代行に関する法律大綱]. https://www.nichibenren.or.jp/library/ja/opinion/report/data/111215_6.pdf. Accessed 15 Sept 2019.

[CR18] Japanese Society of Intensive Care Medicine, Japanese Association for Acute Medicine, and Japanese Circulation Society. 2014. Guidelines for End-of-Life Care in Intensive Care: 3 Recommendations from Academic Societies [Japanese: 集中治療における終末期医療 に関するガイドライン : 3 学会か らの提言]. https://www.jsicm.org/pdf/1guidelines1410.pdf. Accessed 15 Nov 2019.

[CR19] Kai Katsunori (2012). Euthanasia and death with dignity in Japanese law. Journal de Médecine Légale et Droit Médical.

[CR20] Kodama, Mami. 2019. ‘The right to die’ and ‘medical futility,’ the two promoting wheels of dynamic to select and dispose lives deemed ‘unworthy to live or treat [Japanese:「死ぬ権利」と「無益な治療」命の選別と切り捨てへの力動の両輪として]. *Journal of Science and Technology Studies* 17: 55–67. 10.24646/jnlsts.17.0_55.

[CR21] Kondo, Daisuke, and Kentaro Mikami. 2018. 70% of key hospitals halted, avoided life-prolonging care for some terminal patients: Poll. *Mainichi*, 31 May 2018. https://mainichi.jp/english/articles/20180531/p2a/00m/0na/007000c. Accessed 9 Aug 2021.

[CR22] Kumagai Fumie, Ishii-Kuntz Masako (2016). Family violence in Japan: A Life Course Perspective. Singapore: Springer.

[CR23] Lebra, Takie. 2007. The cultural significance of silence in Japanese communication*.* In *Identity, gender, and status in Japan*, 115–126. Folkestone: Global Oriental.

[CR24] Lolich, Luciana, and Kathleen Lynch. 2017. No choice without care palliative care as a relational matter, the case of Ireland. *Soundings* 100 (4): 353–374. 10.5325/soundings.100.4.0353.

[CR25] Mackenzie Catriona, Stoljar Natalie (2000). Relational autonomy : Feminist perspectives on autonomy, agency, and the social self.

[CR26] Masaki, Sakiko, Hiroko Ishimoto, and Atsushi Asai. 2014. Contemporary issues concerning informed consent in Japan based on a review of court decisions and characteristics of Japanese culture. *BMC Medical Ethics* 15: 8. 10.1186/1472-6939-15-8. Accessed 4 Sept 2021. 10.1186/1472-6939-15-8 PMC3923408 24495473

[CR27] Ministry of Health, Labour and Welfare (MHLW). 2018a. Awareness survey on medical care at the final stage of life: Report [Japanese: 人生の最終段階における医療に関する意識調査: 報告書]. https://www.mhlw.go.jp/toukei/list/dl/saisyuiryo_a_h29.pdf. Accessed 9 Oct 2019.

[CR28] Ministry of Health, Labour and Welfare (MHLW). 2018b. End-of-life care: Guidelines for the decision-making process [Japanese: 人生の最終段階における医療・ケアの決定プロセスに関するガイドライン]. https://www.mhlw.go.jp/file/04-Houdouhappyou-10802000-Iseikyoku-Shidouka/0000197701.pdf. Accessed 11 Oct 2019.

[CR29] Ministry of Health Labour and Welfare (MHLW). 2019. Summary report of comprehensive survey of living conditions 2019. https://www.mhlw.go.jp/english/database/db-hss/dl/report_gaikyo_2019.pdf. Accessed 8 Aug 2021.

[CR30] Nakazato Kazuhiro, Shiozaki Mariko, Hirai Kei, Morita Tatsuya, Tatara Ryuhei, Ichihara Kaori, Sato Shinichi, Simizu Megumi, Tsuneto Satoru, Shima Yasuo, Miyasita Mitsunori (2018). Verbal communication of families with cancer patients at end of life: A questionnaire survey with bereaved family members. Psycho-Oncology.

[CR31] Nedelsky, Jennifer. 2012. *Law’s relations: a relational theory of self, autonomy, and law*. New York, NY: Oxford University Press. 10.1093/acprof:oso/9780195147964.001.0001.

[CR32] Nishida, Tsukasa. 1979. *Comparing Japanese-American person-to-person communication: A third culture approach*. PhD dissertation, University of Minnesota.

[CR33] Pugh Jonathan (2020). Autonomy, rationality, and contemporary bioethics.

[CR34] Sandsdalen, Tuva, Reidun Hov, Sevald Høye, Ingrid Rystedt, and Bodil Wilde-Larsson. 2015. Patients’ preferences in palliative care: a systematic mixed studies review. *Palliative Medicine* 29 (5): 399–419. 10.1177/0269216314557882. 10.1177/0269216314557882 25680380

[CR35] Sekimoto Miho, Asai Atsushi, Ohnishi Motoki, Nishigaki Etsuyo, Fukui Tsuguya, Shimbo Takuro, Imanaka Yuichi (2004). Patients’ preferences for involvement in treatment decision making in Japan. BMC Family Practice.

[CR36] Shih Patti, Rapport Frances, Hogden Anne, Bierbaum Mia, Hsu Jeremy, Boyages John, Braithwaite Jeffrey (2018). Relational autonomy in breast diseases care: a qualitative study of contextual and social conditions of patients’ capacity for decision-making. BMC Health Services Research.

[CR37] Tamura Chieko (2006). The family-facilitated approach could be dangerous if there is pressure by family dynamics. Americal Journal of Bioethics.

[CR38] Taylor Shelley E, Sherman David K, Kim Heejung S, Jarcho Johanna, Takagi Kaori, Dunagan Melissa S (2004). Culture and social support: who seeks it and why?. Journal of Personality and Social Psychology.

[CR39] Tonelli Mark R, Misak Cheryl J (2010). Compromised autonomy and the seriously ill patient. Chest.

[CR40] Walter Jennifer K, Friedman Ross Lainie (2014). Relational autonomy: moving beyond the limits of isolated individualism. Pediatrics.

[CR41] Young, Jerome. 2002. Morals, suicide, and psychiatry: A view from Japan. *Bioethics* 16 (5): 412–424. 10.1111/1467-8519.00299. 10.1111/1467-8519.00299 12472089

